# Non-motor Adverse Effects Avoided by Directional Stimulation in Parkinson's Disease: A Case Report

**DOI:** 10.3389/fneur.2021.786166

**Published:** 2022-01-31

**Authors:** Fernando Alonso-Frech, Carla Fernandez-Garcia, Victor Gómez-Mayordomo, Mariana H. G. Monje, Celia Delgado-Suarez, Clara Villanueva-Iza, Maria Jose Catalan-Alonso

**Affiliations:** ^1^Department of Neurology, San Carlos Research Health Institute (IdISSC), Hospital Clínico San Carlos, Madrid, Spain; ^2^Department of Neurosurgery, San Carlos Research Health Institute (IdISSC), Hospital Clínico San Carlos, Madrid, Spain; ^3^Department of Neurology, Hospital Universitario de Móstoles, Madrid, Spain

**Keywords:** non-motor symptoms, deep brain stimulation, subthalamic nucleus, Parkinson's disease, local field potential (LFP), tractography

## Abstract

**Introduction:**

Deep brain stimulation (DBS) is widely used for treatment of advanced, medication-refractory Parkinson's disease (PD). However, a significant proportion of patients may suffer adverse effects; up to 10% will present one or more transient or permanent neurobehavioral events.

**Patient and Methods:**

In our case study, a 44-year-old woman diagnosed with PD 6 years previously who was suffering from motor fluctuations, dyskinesia, and freezing of gait episodes was submitted for DBS and implanted with directional electrodes. Intraoperative local field potentials (LFPs) were recorded. After surgery, conventional monopolar revision was performed. Preoperative 3T MRI studies and postoperative 3D and X-ray data were integrated using the Guide DTI software application (Brainlab), and diffusion tensor imaging tractography traced from cortical areas to each subthalamic nucleus (STN) using Elements software (Brainlab).

**Results:**

We observed that left STN stimulation in the ring mode significantly improved motor symptoms, but the patient presented uncontrollable mirthful laughter. Stimulation was then switched to the directional mode; laughter remained when using the more posteromedial contact (3-C+) but not 2-C+ or 4-C+ at the same parameters. Interestingly, LFP recordings showed the highest beta-band activity over contacts 4 and 2, and very scarce beta power over contact 3. The orientation of the directional leads was selected based on the 3D postoperative X-rays. Associative fibers showed the shortest distance to contact number 3.

**Conclusion:**

Stimulation of the STN can affect motor and associative loops. The use of directional electrodes is a good option to avoid not only undesirable capsular or lemniscal effects, but also limbic/associative events. Oscillatory activity in the beta range that preferentially takes place over the somatomotor STN region and is closely related to motor improvement, provides a reliable guide for optimizing the DBS programming. The importance of the exact location of electrical stimulation to determine the non-motor symptoms such as mood, apathy, attention, and memory, as well as the usefulness of biological markers such as LFP for optimal programming, is discussed in relation to this case.

## Introduction

Deep brain stimulation (DBS) of the subthalamic nucleus (STN) is a safe and effective treatment for motor symptoms in advanced Parkinson's disease (PD) (1). However, up to 50% of patients may suffer adverse effects (2) and 10% could potentially present one or more transient or permanent neurobehavioral events (3).

Complications associated with STN-DBS can be grouped between those derived from the surgical procedure and those from stimulation, such as the spread of the volume of tissue activated (VTA) through the boundary areas of the STN (e.g., internal capsule, medial lemniscus). Even with optimal lead placement in the somato-motor region of the STN, the electrical current could expand through limbic or associative regions, causing clinical or subclinical non-motor symptoms.

Over the last few years there has been a growing interest in the neuropsychiatric effects of STN-DBS. Prospective studies have described cases of mood changes and behavioral disturbances (4–11), as well as the influence of STN-DBS on fatigue, impulse control disorder, and weight gain in prospective studies (12–14). The frequency of a negative impact of DBS on non-motor symptoms varies according to studies (15–19). For example, in the review from Temel et al. (20) 41% of 1,389 patients who underwent bilateral STN-DBS presented an impairment in executive functions, 8% exhibited major depression symptoms, and 4% showed signs of hypomania. Although it is unnecessary to remove the device in most of these cases, non-motor symptoms could negatively impact on patients' quality of life (21, 22).

Conventional cylindrical electrodes for DBS create a radial current diffusion in the horizontal plane of the lead. Newly developed directional electrodes are based on the classic design of a quadripolar DBS lead, but the two middle electrode levels are segmented into three contacts, each spanning ~120° of the circumference. If all segments are activated together, a ring electrode is simulated, and a corresponding spherical VTA is generated (omnidirectional stimulation). By activating only one segment as a cathode, the VTA can be shaped in the horizontal plane and the current will be injected in a preferential angular direction (23). This preferential directional stimulation prevents the VTA from expanding into adjacent eloquent structures like the cortico-spinal tract and the medial lemniscus, and avoids associated adverse effects such as muscle twitches and paresthesia, respectively. They could also potentially be helpful in preventing non-motor symptoms in STN-DBS stimulation. Furthermore, it is important to have extensive knowledge of neurophysiological biomarkers of PD and neuroimage techniques to facilitate outlining specific neuronal circuits for directional programming, improve target accuracy, and adapt DBS treatment to patient-specific symptoms.

We present the case of a 44-year-old woman who developed uncontrollable mirthful laughter during left STN stimulation after ring stimulation, which stopped when switched to directional stimulation.

## Case Description

The patient had a personal history of dyslipidemia, anxiety-depressive syndrome, and a family history (cousin) of PD. Symptoms began at the age of 38 years with pain in the left leg and dystonic posture of the big toe. It was difficult for the patient to go up- and down-stairs and get up from a seat. The patient took more than a year to consult for these symptoms but was finally diagnosed with PD 2 years later, and improved significantly with oral levodopa treatment.

The patient attended our hospital at the age of 42 years. By that time, patient was on immediate-release levodopa-carbidopa-entacapone (150/37.5/200 mg) four times a day; extended-release levodopa-carbidopa (200/50 mg) at night; safinamide (50 mg) and pramipexole extended-release (2.1 mg) once a day. The patient suffered motor fluctuations with delayed response to every levodopa dosage (60–120 min), marked wearing-off phenomena, and some dose failures. During the OFF state, patient was unable to walk due to severe freezing of gait episodes and occasional falls. The ON states (~2–3 h) were functionally optimal, and the patient could take care of the housework and their children, although with severe axial and limb dyskinesia (Time course of symptoms and interventions in Supplementary Figure 1).

The Unified Parkinson's Disease Rating Scale (UPDRS) score in the OFF state was 34, and the Up and Go test was impossible without a walker-aid, took 54 s, and presented severe freezing during turns. The ON state was reached after 45 min of taking levodopa (300 mg), the UPDRS score was 8, and the Up and Go test took 12 s without freezing. Moderate axial dyskinesia was present. The brain MRI did not show any abnormalities. The neuropsychological evaluation showed no decline in cognitive domains, with a Mattis Dementia Rating Scale score of 139/144 (attention 36, initiation/perseveration 35, construction 6, conceptualization 38, memory 24). However, the patient scored high in self-reported behavioral questionnaires for screening of anxiety and depression.

Treatment was optimized with subcutaneous apomorphine injections (3 mg) on-demand; a few weeks later, the patient was submitted for DBS. Target coordinates were obtained by merging preoperative 3T MRI sequences with preoperative CT stereotactic images (Leksell Frame G, Elekta, Crawley, UK). The patient underwent awake surgery, after overnight withdrawal of dopaminergic medication (OFF medication). Sedation with dexmedetomidine was used but discontinued during microelectrode recordings (MER). Three microelectrodes were used simultaneously according to the calculated target coordinates: one central, one 1.5 mm lateral, and one 1.5 mm anterior to the center. MER was started (−10 mm) above the calculated target and progressed in 0.5 mm steps or continuous recording if the single-neuron activity was present. Emphasis was placed on defining the dorsolateral region of the STN (which corresponds to the motor segment of the nucleus), characterized by the presence of outstanding single-neuron activity and driving responses to active/passive limb movements. Microstimulation was performed at the level of each trajectory where the prominent neuronal activity or driving was recorded and at the theoretical target point (0 mm), with clinical assessment of both, such as therapeutic benefits and the presence of adverse effects. The best track was then selected. The final objective was to place the segmented levels of the macroelectrode (total length 4 mm) so that they coincided with the levels of the trajectory where the MER and microstimulation would be most favorable.

**Figure 1:**
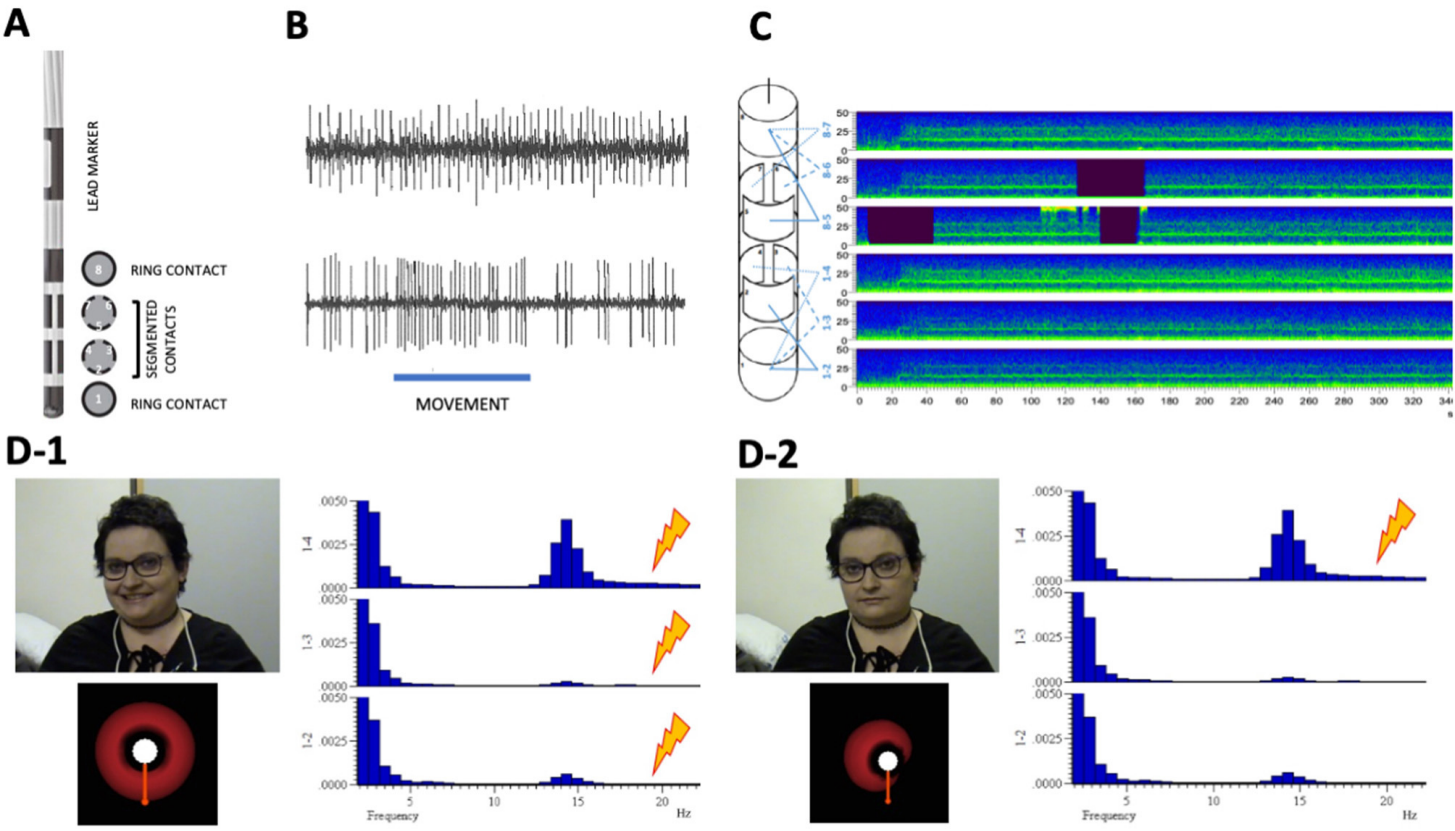
**(A)** Directional lead, **(B)** microelectrode recordings (MER), **(C)** local field potential (LFP) recordings, and **(D)** adverse effects by type of stimulation according to neurophysiological findings. **(A)** Longitudinal and axial scheme of the directional lead, showing the lead marker and the four contact levels: the two middle levels are split into three segmented contacts, spanning 120 degrees (1-2-3 more ventral and 3-4-5 more dorsal) and the highest (8) and lowest (1) contacts are ring-shaped. **(B)** Sample of MER from patient. Up: Spontaneous subthalamic nucleus (STN) spike activity. Down: STN spike activity during passive wrist extension movement (underlined), showing the driving effect. **(C)** LFP recording. Left: Scheme of bipolar montage between the lowest ring electrode and every inferior segmented contact level and between the highest ring electrode and every superior segmented contact level. **(C)** Right: Example of a bipolar LFP recording (sonogram mode) during 340 s (X-axis), with presence of activity in the beta band (13–35 Hz) in all the bipolar derivations (Y-axis), more prominent in derivation 1–4. **(D)** Adverse effects by type of stimulation. **(D-1)** Up left: Ring mode stimulation elicited mirthful laughter in the patient. Down left: Axial representation of theoretical volume of tissue activation (VTA) in ring mode stimulation [2-3-4]—C+. Right: Fast fourier transformation of intraoperative STN activity recorded from the left macroelectrode (bipolar montage 1-2, 1-3, 1-4), showing a clear beta (14.6 Hz) peak over contact (4). **(D-2)** Up left: Directional stimulation (4)—C+ did not elicited adverse effects in the patient. Down left: Axial representation of theoretical volume of tissue activation (VTA) in directional mode stimulation (4)—C+. Right: Fast fourier transformation of intraoperative STN activity recorded from the left macroelectrode (bipolar montage 1-2, 1-3, 1-4), showing a clear beta (14.6 Hz) peak over contact (4).

Directional DBS leads (Cartesia; Boston Scientific, Valencia, California) were bilaterally implanted. These leads have four contact levels: the two middle levels are split into three segmented contacts, spanning 120 degrees, and the highest and lowest contacts are ring-shaped (Figure 1A). The final position of the electrode and segmented contacts were determined according to best the MER (outstanding single-neuron activity and driving responses to active/passive limb movements) (Figure 1B). Once the directional leads were implanted and fixed (electrode mark facing the anterior position), local field potentials (LFPs) were recorded for 300–500 s using a custom-made external connection. We used a bipolar montage between the lowest ring electrode and every inferior segmented contact level, and between the highest ring electrode and every superior segmented contact level (Figure 1C). LFPs were amplified 10,000-fold and filtered at 1–3,000 Hz (D-150, Digitimer, Cambridge, UK). Signals were digitized at 10,000 Hz by an analog/digital converter (1401 plus, Cambridge Electronic Design, Cambridge, UK) connected to a personal computer (PC). Segments with artifacts were discarded, and the remaining segments were available for off-line analysis. LFP recordings aim to assess oscillatory activity in the beta frequency range (13–35 Hz) on each contact. Beta oscillations are mainly recorded on the somato-motor region of the STN (24). Beta activity is also well-correlated with rigidity and bradykinesia (25) and decreases after levodopa uptake (26) and when turning DBS ON (27). For all of these reasons, beta activity is considered a reliable biological marker of the parkinsonian state and can help to optimize DBS programming (28, 29).

**Figure 2:**
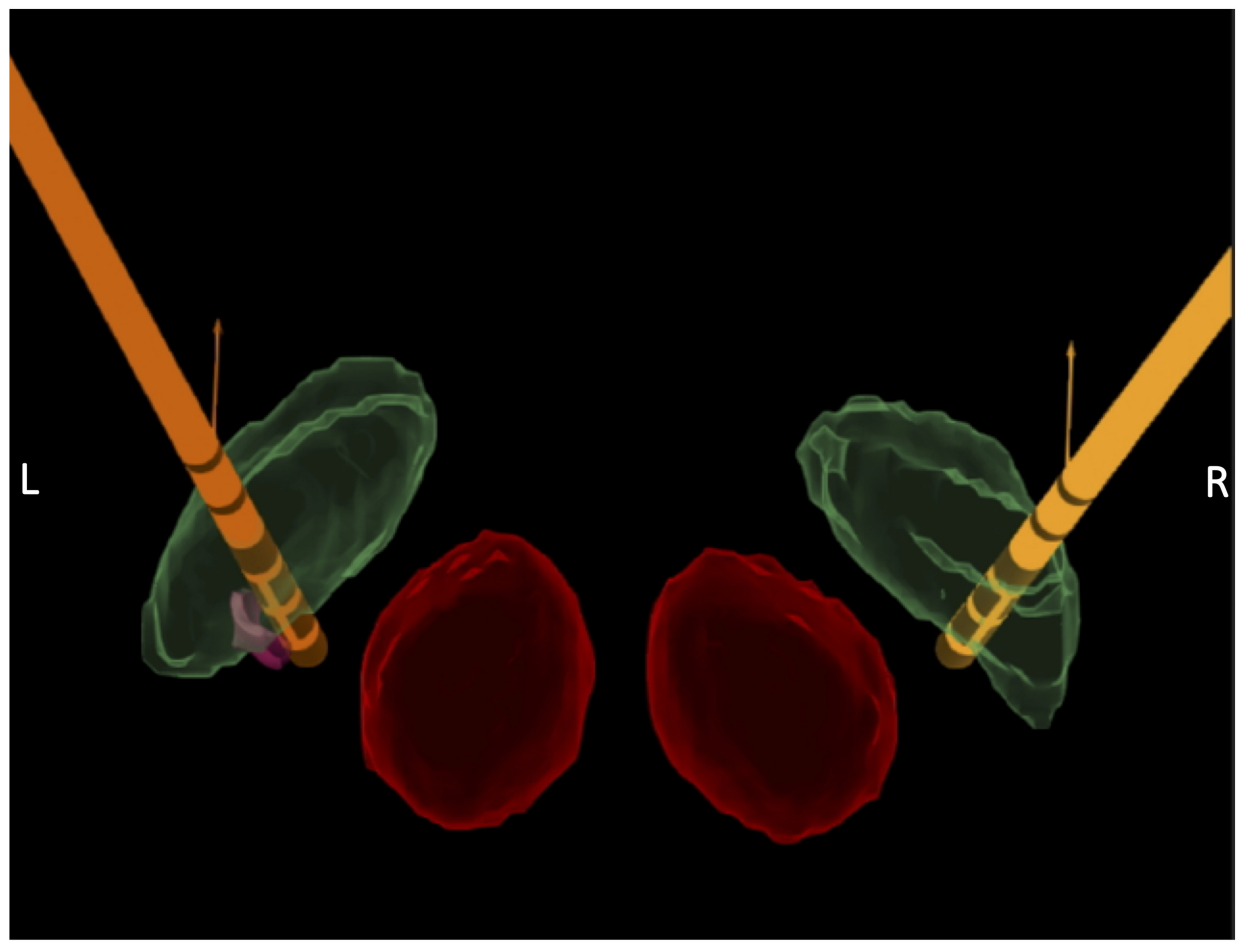
Lead location confirmation. Fusion of postoperative CT scan with preoperative 3T axial MRI, using Elements^©^ software (Brainlab^©^) and Guide XT^©^ software (Boston Scientific, Valencia, California), showing the final position of bilateral directional leads (orange) within the subthalamic nucleus (STN; green) and the red nucleus (red). The left lead was slightly medialized inside the STN and a small volume of tissue activated (VTA; pink sphere) was generated close to contact 3, to highlight its position in the ventromedial region of the nucleus.

Postoperative stereotactic CT was performed to ensure no surgical complications, and finally, the electrode wires were internalized and a pulse generator was implanted. Optimal lead location was confirmed by fusing postoperative 3D CT and preoperative 3T MRI, and 3D X-ray was used to assess lead orientation (Figure 2). Preoperative 3T MRI and postoperative 3D and X-ray data were integrated into the Guide XT Software (Boston Scientific) and Elements Software (Brainlab, Munich, Germany) to evaluate the lead position in the STN and conduct tractographic analysis.

## Diagnostic Assessment

On day 12 after surgery, a standard monopolar review was performed to assess the contact with the best therapeutic window (TW), which was defined for each lead contact as the difference between the adverse effect threshold (AET; the minimum current that induced side effects, or 5 mA) and the efficacy threshold (ET; the minimum current that induced rigidity reduction). If two contacts shared the same TW, other parameters such as developing dyskinesia were considered for the selection. For the right STN, contacts 3 and 6 (posterolateral) shared the widest TW (3 mA). For the left STN, contact 4 (posteromedial) had the widest TW (2 mA), but contacts 2 (anterior) and 3 elicited dyskinesia at lower intensities. Neurophysiological findings showed higher beta power at 14.7 Hz over contact 3 on the right STN and at 14.6 Hz over contact 4 on the left STN (Figure 1D). For these reasons, the left STN was initially stimulated in the ring mode, with contacts [2-3-4] as a cathode. The right STN was stimulated with contact 3 as a cathode. Stimulation parameters were set at current 1.5 mA, pulse width 60 μs, and frequency 130 Hz.

During the following 3 weeks, the patient improved significantly in their activities of daily living and barely presented freezing of gait or wearing-off episodes. The patient reported that their relatives said they talked and laughed more than usual, and patient did the housework with uncommon energy. Approximately 1 month later at the clinic, an increase in current intensity provoked uncontrollable mirthful laughter in the patient. We decided to perform another monopolar review and observed that the mirthful laughter was triggered by left STN ring stimulation [2-3-4] (Supplementary Video 1). When stimulation was switched to the directional mode, the laughter remained with contact [3-C+] but not with contacts [2-C+] or [4-C+] using the same parameters.

Stimulation was set at directional [4-C+] stimulation. To date, the patient has not presented with mirthful laughter again (Supplementary Video 2). Motor improvement was maintained over the following months. At 12 months, the UPDRS-III score in OFF was 8 (−76%). The patient performed the Up and Go test in 24 s in 11 steps without freezing (patient was previously unable to walk autonomously). Medication was reduced from 985 mg of levodopa-equivalent dose preoperatively to 605 mg post-DBS (−39%).

**Figure 3:**
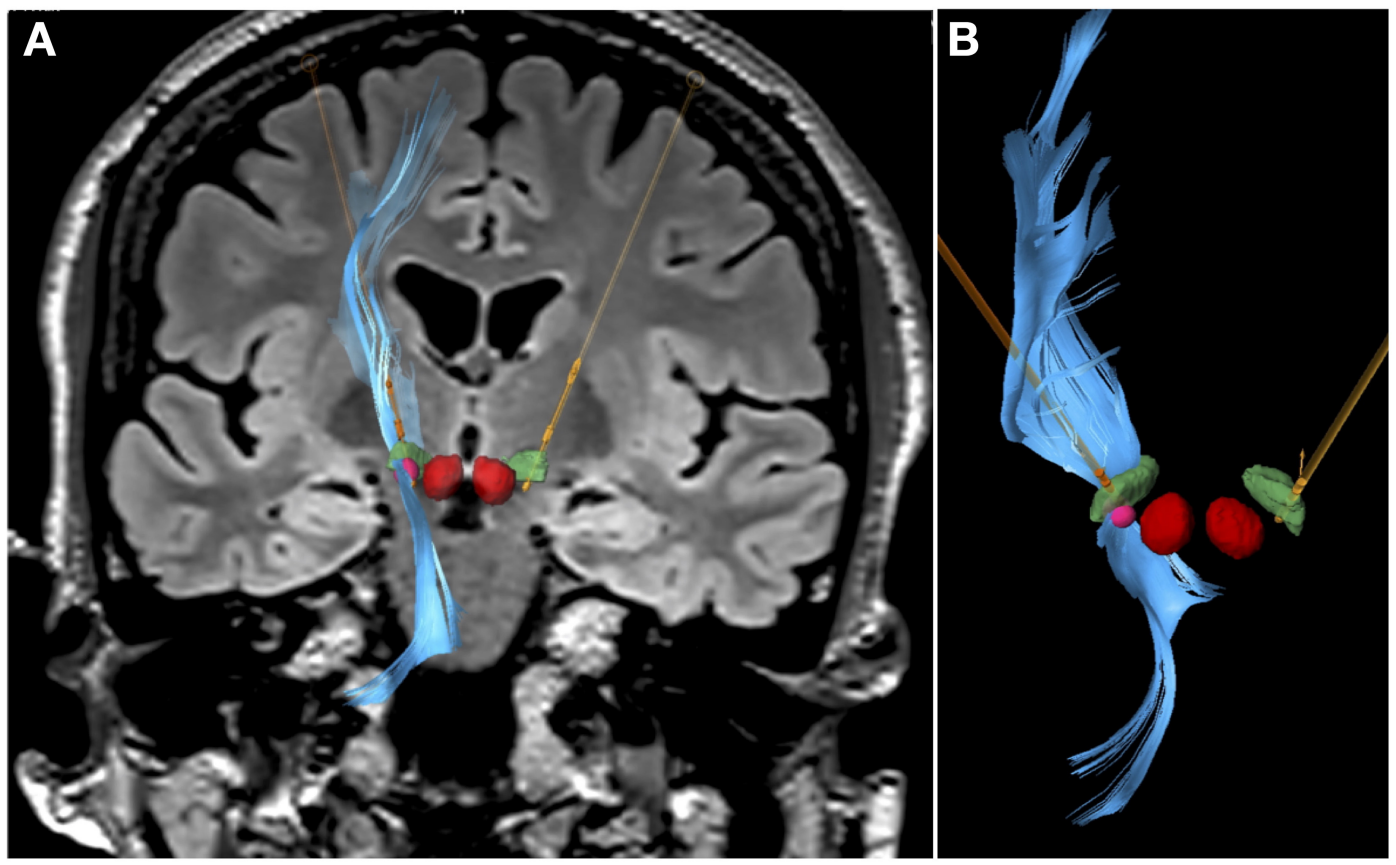
Tractography study. Representation of the associative fibers stimulated when contact 3 in the left STN was activated at 1.5 mA, 60 μs, and 130 Hz. **(A)** Coronal flair 3T MRI view, with segmentation of bilateral red nucleus (red), bilateral STN (green), directional leads (orange), and VTA (pink sphere) generated with contact 3 stimulation. Fibers toward the prefrontal cortex in the left side are represented in blue. **(B)** Close view of the left directional lead, left STN and the fibers passing through the VTA generated (in contact 3) toward the frontal region. Images obtained with Elements^©^ software (Brainlab^©^) and Guide XT^©^ software (Boston Scientific).

In the tractography study, regions of interest were manually segmented in the prefrontal cortex to depict the associative tract, in the midbrain nuclei and accumbens nucleus to depict the medial forebrain bundle, and in the fornix and cingulum to depict the limbic circuit. Fibers were restricted to those passing through the left STN. Associative fibers showed the shortest distance to contact number 3 (Figure 3).

## Discussion

To our knowledge, this case represents the first to describe how steering stimulation using directional leads can avoid non-motor adverse effects (laughter and hypomania) in STN-DBS therapy.

Directional DBS was initially conceived to expand the TW by increasing the AET (30, 31). However, complications of DBS may also occur if current leaks toward other regions inside the STN. Given that STN is a small structure of only 10 mm in diameter (32) and is functionally heterogeneous, the current stimulation necessary to improve motor symptoms can easily diffuse in undesired areas. The STN is divided into three subterritories (somato-motor, associative, and limbic) according to the functionally segregated connections that it receives from the striatum and globus pallidus (33–35). In addition to the cortico-striatal input, the STN also receives projections from the frontal cortex through the hyperdirect pathway (36), also somatotopically organized. This strategic anatomical-functional disposition of the STN provides a leading role in the control and integration of motor, cognitive, and emotional aspects that modulate cortico-striatal processing (37–39)—playing a key role in the inhibitory control of behavior, supported by electrophysiological evidence (40) and functional neuroimaging (41). Traditionally, the somato-motor region is located in the dorsal region of the STN; the limbic region is more ventral, and the associative region is located between both. However, it is known that, unlike the striatal territories, the borders between the functional territories within the STN can overlap (33). Moreover, the high degree of convergence and overlap between projections from functionally diverse cortical areas, and the stimulation of passing fibers from each functional cortical region that travel widely through the STN (33), may underlie non-motor side effects seen in DBS for PD.

For these reasons, it is likely that despite optimal electrode placement in the somato-motor region of the STN, current diffusion to other subregions of the nucleus, such as limbic or associative, may cause clinical effects of non-motor type during chronic stimulation of the STN. These non-motor side effects have been reported as case reports describing changes in mood and behavior (4–11), as well as prospective studies showing the influence of DBS in fatigue, apathy, depression, anxiety, impulse control disorder, and weight gain. The reported frequency of these complications varies (15–18).

Physiological laughter occurs in an appropriate social context and is accompanied by an emotional feeling of mirth. In contrast, pathological laughter consists of uncontrollable outbursts of laughter that are inappropriate for the external circumstances and emotional state of the patient (42). There has been a wide variety of case reports in the last century showing that pathological laughter can appear in many neurological diseases. It has been described as the prodromal of a stroke (43), pseudobulbar palsy (44), strategic cerebral lesions or diffuse cerebral disease (45), and epileptic disorders such as temporal lobe epilepsy or hypothalamic hamartomas (46). Due to the huge variety of possible regions reported, it has been postulated that this symptom is the consequence of a dysfunctional “cortico-limbic-subcortical-thalamo-ponto-cerebellar network” (42). Recently, it has been hypothesized that two circuits might interact: an “emotional” system that exerts excitatory control (temporal and frontal lobes, basal ganglia, thalamus, and hypothalamus) and a “volitional” system that generates inhibitory control (lateral premotor cortices). Both systems project to the periaqueductal gray matter for the final coordination of the facial, respiratory, and vocal components of laughter (42).

As previously mentioned, the STN has several functional regions, such as the dorsolateral area for motor control, the central area for associative/cognitive tasking, and the ventromedial region, which projects to limbic circuits (47). The medial tip of the STN, in close anatomical relationship with the lateral hypothalamus, is markedly innervated by the dorsal anterior cingulate cortex, the ventromedial prefrontal cortex/orbitofrontal cortex (33), and the nucleus accumbens (*via* the medial forebrain bundle) (48). The electrical stimulation of these projections, either by an epileptic focus or by the effect of DBS, could cause an overstimulation of the excitatory emotional system that produces pathological laughter (49). However, the dysregulation of the volitional inhibitory system (which projects to premotor regions) may also explain the cases reported by the stimulation of dorsal regions of the STN (4).

Mirthful laughter due to acute STN-DBS has been previously described and attributed to stimulation of the limbic and associative loops (4, 50, 51). In these case reports, the patients were treated by conventional DBS leads, and the symptoms were related to increasing stimulation parameters. Incoercible and unappropriated laughter in our patient may have occurred due to associative fibers activation when monopolar directional stimulation was applied on contact 3. A considerable degree of inter-individual variability for motor and non-motor outcomes in STN-DBS treatment (52, 53) is related to the position of active DBS contacts (53). Therefore, the neuropsychological impairment and the likelihood of neuropsychiatric side-effects could be reduced by focusing the stimulation toward the dorsal border (54) or the motor sub-region of the STN (55).

Mosley et al. (56) found that although most non-motor symptoms generally improve after STN-DBS, the improvement in mood/apathy, attention/memory, and sleep is dependent on the exact location of electrical stimulation. In this study, the voxels that most reduced mood/apathy inside the STN were located in the sensorimotor STN. This sub-region is also part of the posterior dorsal STN. The stimulation of the most posterior contact in our patient induced mirthful laughter and some characteristic hypomanic symptoms, such as chatterbox behavior and hyperactivity in daily activities. The direct effect of neurostimulation over the associative region of STN by contact 3 may be supported by the electrophysiological findings, since this contact exhibited very low beta power and, interestingly, a narrow TW. Conversely, contact 4 showed the highest beta activity and the widest TW, which would mean it was specifically located over the somato-motor area. A clear relationship between beta activity and the somato-motor region of STN has been demonstrated by many studies (24, 57, 58).

The tractography study in our patient showed the shortest distance between associative fibers to contact 3. This supports the notion that stimulation of specific areas of the nucleus may modulate connectivity within associative and limbic circuits of the basal ganglia (58–62). However, tractography findings must be interpreted with caution since this technique presents inherent limitations. Tractography results need complementary validation, as they could be those derived from neurophysiological data.

## Conclusions

Biomarkers such as LFP recordings from implanted macroelectrodes, in concurrence with tractography findings, could provide a unique opportunity to guide neurostimulation toward more convenient motor regions and thus avoiding non-motor adverse effects. The use of directional leads may lead us closer to precision-personalized medicine.

## Data Availability Statement

The original contributions presented in the study are included in the article/Supplementary Material, further inquiries can be directed to the corresponding author.

## Ethics Statement

Ethical review and approval was not required for the study on human participants in accordance with the local legislation and institutional requirements. The patients/participants provided their written informed consent to participate in this study. Written informed consent was obtained from the individual(s) for the publication of any potentially identifiable images or data included in this article.

## Author Contributions

FA-F and CF-G were involved in the conception and design. FA-F, CF-G, VG-M, and MM were involved in the acquisition, analysis, and interpretation of the data. FA-F wrote the first draft of the manuscript. FA-F, CF-G, and VG-M wrote the sections of the manuscript. MM, CD-S, CV-I, and MC-A contributed to manuscript revision, read, and approved the submitted version. All authors contributed to the article and approved the submitted version.

## Conflict of Interest

The authors declare that the research was conducted in the absence of any commercial or financial relationships that could be construed as a potential conflict of interest.

## Publisher's Note

All claims expressed in this article are solely those of the authors and do not necessarily represent those of their affiliated organizations, or those of the publisher, the editors and the reviewers. Any product that may be evaluated in this article, or claim that may be made by its manufacturer, is not guaranteed or endorsed by the publisher.

## Supplementary Material

Supplementary Video 1 Mirthful laughter during ring-mode stimulation.

Supplementary Video 2 No emotional changes in patient during segmented stimulation.

Supplementary Figure 1 Time course of symptoms and interventions. Values indicate years. M, months.
