# Supplementary figures and images for: Non-motor Adverse Effects Avoided by Directional Stimulation in Parkinson's Disease: A Case Report
Source: Front Neurol. 2022 Jan 31;12:786166. doi: 10.3389/fneur.2021.786166 (PMC8843015; doi:10.3389/fneur.2021.786166)

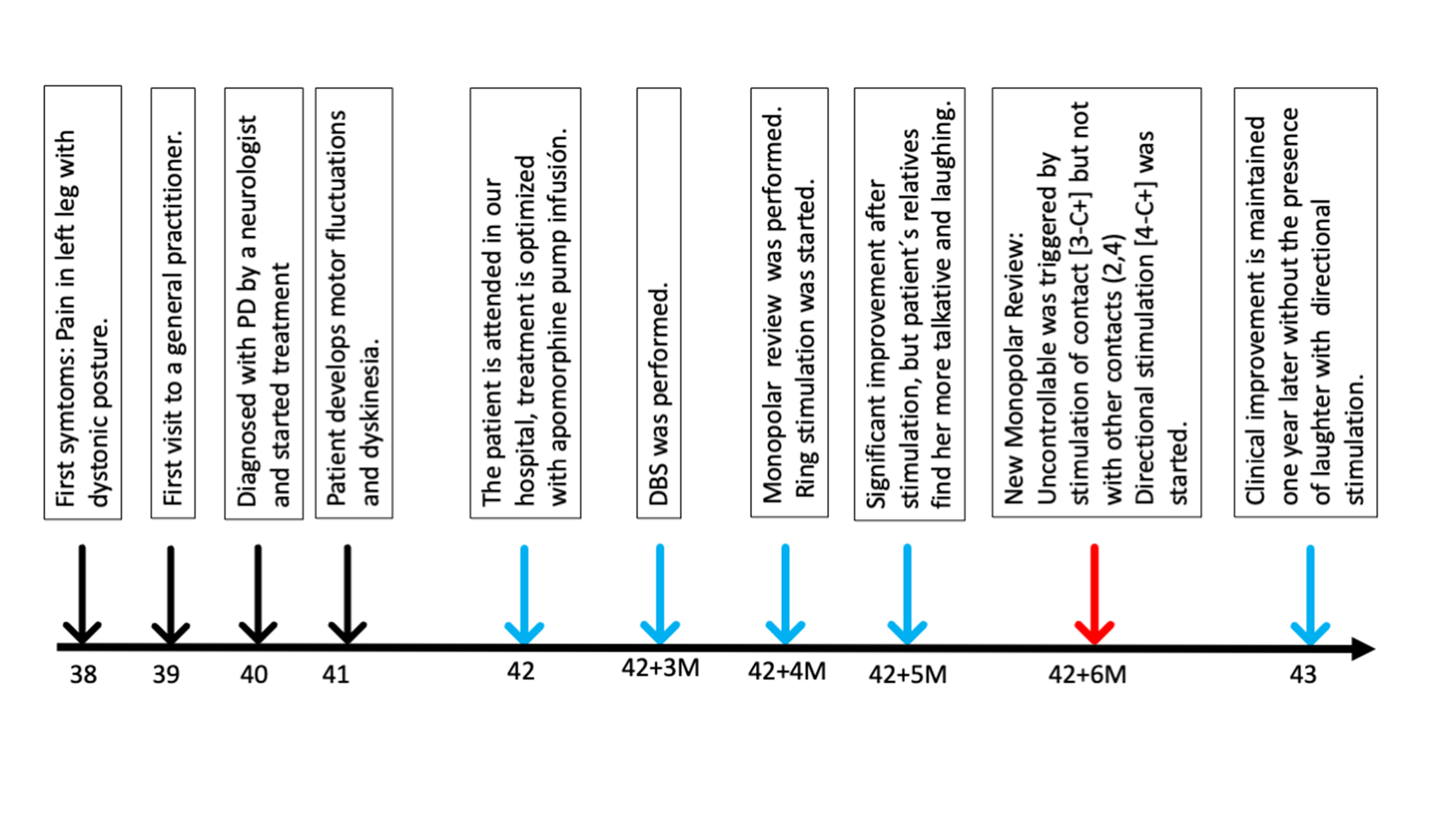

Supplement: Supplementary Figure 1 — Time course of symptoms and interventions. Values indicate years. M, months. [file Image_1.JPEG]
